# Improvements in Behavioral Symptoms following Antibiotic Therapy in a 14-Year-Old Male with Autism

**DOI:** 10.1155/2013/239034

**Published:** 2013-06-19

**Authors:** P. Lucas Ramirez, Kelly Barnhill, Alan Gutierrez, Claire Schutte, Laura Hewitson

**Affiliations:** ^1^The Johnson Center for Child Health and Development, Austin, TX 78701, USA; ^2^Department of Psychiatry, University of Texas Southwestern, Dallas, TX 75235, USA

## Abstract

This case report describes the benefits of antibiotic and antifungal therapy on behavior in a child with autism undergoing treatment for encopresis. Over the course of treatment, the child exhibited a reduction in aberrant behaviors, increased gastrointestinal function, and improved quality of life.

## 1. Introduction

A fourteen-year-old male child with autism was evaluated at our center following a nine-month history of encopresis. His gastrointestinal history included diarrhea for the first two years of life, related to possible soy formula intolerance. Reportedly, stool consistency improved dramatically at the age of four when gluten, casein, and soy proteins were removed from his diet. At seven years of age, casein was reintroduced, resulting in reported negative and self-stimulatory behaviors and disrupted sleep. After two weeks, casein was once again removed. Gluten was reintroduced six months later. Negative responses, including self-stimulatory behaviors, aggression, and self-injurious behaviors, were reported shortly after these dietary changes. Gluten was once again eliminated from his diet. Over the next few years, dietary elimination became less stringent until there were no dietary interventions or restrictions in place. At eleven years of age, his aggression increased, and the elimination of gluten and casein was again implemented, which the parents reported helped with the management of his behavioral escalations.

The use of pharmaceutical agents for behavioral control began when the child was six years old with risperidone and clonidine. At the age of thirteen, valproic acid was added when his aggressive behaviors escalated, made apparent by aggression directed toward his mother. Only mild reductions in negative behaviors were attributed to the use of these medications.

For nine months prior to his first appointment at our clinic, the patient was soiling his clothes with daily bowel movements. He was also complaining of pain associated with bowel movements. His stool was reportedly sticky, unformed, and extremely foul-smelling. There were no medical treatment or dietary changes at that time. He was evaluated for encopresis by a pediatric gastroenterologist. An abdominal X-ray did not show constipation, and his physical examination was benign; no treatment options were offered. Constipation management prescribed by a subsequent pediatric gastroenterologist was unsuccessful in resolving the encopresis.

## 2. Case Presentation

At his initial visit to our clinic, at age 14, the patient was evaluated for ongoing encopresis. His physical examination was unremarkable. He appeared to be anxious, initially regulating through the use of physical self-stimulatory behaviors. As the appointment progressed, the patient's stimulatory behaviors decreased and he independently used a communication device, spelling out his needs and feelings.

A Comprehensive Parasitology (CP, Doctor's Data, Inc. St. Charles, IL, USA) was performed on a stool sample to screen for possible overgrowth of pathogenic bacteria. The results of the CP revealed an appropriate amount of beneficial bacteria, although no *Enterococcus* spp. was cultured. Commensal bacteria consisting of both alpha and gamma hemolytic *Streptococcus* spp. and hemolytic *Escherichia coli* were present. *Clostridium* spp. was cultured at a 3+ growth. The fungal culture revealed a 3+ growth of *Geotrichum klebahnii*, which was reported to be resistant to fluconazole and itraconazole. Microscopic inspection of the stool was negative for yeast or parasites and the immunoassay for *Giardia* and *Cryptosporidium* was negative as well. A ten-day course of metronidazole and a subsequent twenty-day course of ketoconazole were prescribed for management of the *Clostridium* spp. and *G. klebahnii*, respectively. Approximately 20 days after the initiation of the metronidazole (ten days into the ketoconazole course), the patient's encopresis resolved. His stool consistency improved and he no longer complained of pain associated with bowel movements. Parental reports indicated that his aggressive and self-injurious behaviors significantly improved and his self-stimulatory behaviors decreased dramatically during the course of the treatment.

The benefits from the metronidazole and ketoconazole were not permanent. The encopresis resumed and the aggressive and self-injurious behaviors increased during the first two weeks following the completed courses of medications. Ketoconazole was repeated but did not produce the same benefits in gastrointestinal symptoms as was previously reported. This was, therefore, followed by another course of metronidazole. With continued treatment, it was recognized that the gastrointestinal and behavioral benefits appeared to be associated with the use of this antibiotic. Antifungal medications alone did not resolve symptoms. Multiple courses of metronidazole were, therefore, used over the next two years to address the recurrence of symptoms between treatments. Resolution of the encopresis and pain associated with bowel movements was observed approximately ten days after completion of each course. Episodes of aggression and self-injurious behavior that occurred in between treatments included the child putting his head through a wall at home, biting himself and his family, breaking a teacher's nose with a head butt, and breaking the rear windshield of a vehicle by head banging. During treatment, the patient's parents also reported a decrease in aberrant behaviors, as well as an increase in developmental skills including independent toileting, initiation of preferred activities, and improvements in his ability to independently manage his behavior and decision-making. Although the improvements in behavior were based on parental report, during subsequent office visits, the patient communicated a strong desire to continue treatment due to improvements in his health and quality of life.

## 3. Discussion

This short report highlights the beneficial response to antibiotic treatment as evidenced by the management of encopresis and improvements in aberrant behaviors. For this patient, repeated treatment with antibiotics greatly improved gastrointestinal function, decreased reported bowel pain, and reduced aggressive and self-injurious behaviors.

The presence of bacteria, such as *Clostridia* spp., may populate the gastrointestinal tract of children with autism at much higher rates than control children [1], and it has been suggested that antibiotics may improve behavioral symptoms in some children with autism [2, 3]. Here, we report that this patient's quality of life improved after treatment with antibiotics. Questions that have yet to be answered pertain to the lack of long-term benefits from the use of antibiotics and concerns over their long-term use, as well as the mechanism of gastrointestinal dysbiosis on neurological functioning. Further research is needed to address these questions.
